# Supplementary material for: Structural and Functional Insights into Human Nuclear Cyclophilins
Source: Biomolecules. 2018 Dec 4;8(4):161. doi: 10.3390/biom8040161 (PMC6315705; doi:10.3390/biom8040161)
Supplement: Supplementary file 1 [file biomolecules-08-00161-s001.zip › biomolecules-389905-SI/biomolecules-389905_supplementary Figure S1.pdf]

A)

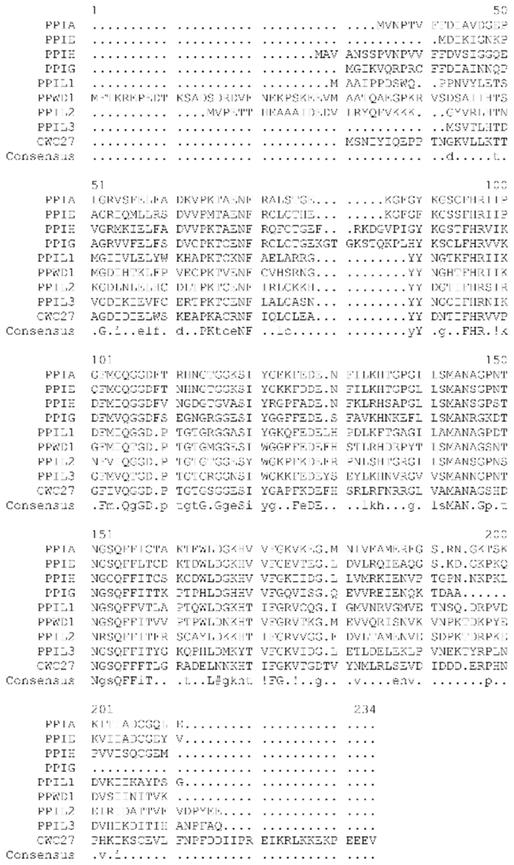

B)

	% ident	%sim	RMSD (Å)	notes	residues in alignment
**PPIH**	54	69	0.477	cyclophilin only	9-177
**PPIE**	68	82	0.406	cyclophilin only	1-156
**PPIL1**	51	67	1.604	NMR structure	12-139
**PPIL2**	50	61	0.699	cyclophilin only	35-161
**PPIL3**	48	61	0.731		9-144
**CWC27**	43	61	0.797	cyclophilin only	22-133
**PPWD1**	49	59	0.814	cyclophilin only	38-184
**PPIG**	57	66	0.46	cyclophilin only	8-155

C)

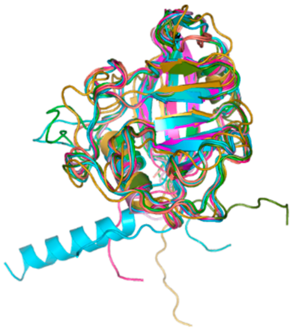

Figure S1. Alignment of the spliceophilins against the canonical cyclophilin PPIA. (**A**) Sequence alignment as performed by the MULTALIN server. (**B**) Quantification of % identity and % similarity across the primary sequence of the cyclophilin domains as calculated in pairwise alignment within BLASTp. Also, quantification of structural alignment (RMSD) between each spliceophilin structure and PPIA as reported by pyMOL. (**C**) Visual representation in cartoon format of a representative of each spliceophilin structure aligned with PPIA.
